# Voltammetric Determination of Acetaminophen and Tryptophan Using a Graphite Screen Printed Electrode Modified with Functionalized Graphene Oxide Nanosheets Within a Fe_3_O_4_@SiO_2 _Nanocomposite

**Published:** 2019

**Authors:** Hadi Beitollahi, Fariba Garkani-Nejad, Somayeh Tajik, Mohammad Reza Ganjali

**Affiliations:** a *Environment Department, Institute of Science and High Technology and Environmental Sciences, Graduate University of Advanced Technology, Kerman, Iran. *; b *Department of Chemistry, Graduate University of Advanced Technology, Kerman, Iran.*; c *NanoBioElectrochemistry Research Center, Bam University of Medical Sciences, Bam, Iran. *; d *Center of Excellence in Electrochemistry, University of Tehran, Tehran, Iran. *; e *Biosensor Research Center, Endocrinology & Metabolism Molecular-Cellular Sciences Institute, Tehran University of Medical Sciences, Tehran, Iran.*

**Keywords:** Acetaminophen, Tryptophan, Nanocomposite, Graphene, Graphite screen printed electrode

## Abstract

A high sensitive electrochemical nanostructure sensor based on graphene oxide/Fe_3_O_4_@SiO_2_ nanocomposite modified graphite screen printed electrode (GO/Fe_3_O_4_@SiO_2_/SPE) has been developed for trace analysis of acetaminophen. The electrochemical study of the modified electrode, as well as its efficiency for simultaneous voltammetric oxidation of acetaminophen and tryptophan is described. Compared with bare SPE the GO/Fe_3_O_4_@SiO_2_/SPE exhibited excellent electrocatalytic activity toward the oxidation of acetaminophen. The plot of catalytic current versus acetaminophen concentration showed a linear segment in the concentration range 0.5 to 100.0 µM. The detection limit of 0.1 µM was obtained using calibration plot. Also the anodic peaks of acetaminophen and tryptophan in their mixture can be well separated. The GO/Fe_3_O_4_@SiO_2_/SPE has been successfully applied and validated by analyzing acetaminophen and tryptophan in urine and pharmaceutical samples.

## Introduction

Acetaminophen (N-acetyl-p-aminophenol or paracetamol), is a long-established substance being one of the most extensively employed drugs in the world (1). Acetaminophen is the most widely used analgesic and antipyretic drug in the world. Acetaminophen is useful in the treatment of cold, cough, and asthma (2). Hence, acetaminophen is the major ingredient in cold and influenza medications (3). It is also non carcinogenic and an effective substitute for aspirin for the patients who are sensitive to aspirin and safe up to therapeutic doses. Limited use of acetaminophen does not exhibit any harmful side effects, while overdosing and chronic use produces accumulation of its toxic metabolites that may cause kidney and liver damage (4). To date, a variety of methods such as high performance liquid chromatography (HPLC), spectrofluorimetry, liquid chromatography/electrospray mass spectrometry, spectrophotometry, and electrochemical methods, such as potentiometry, polarography, and voltammetry have been developed for the determination of acetaminophen (5-11). Acetaminophen is electro-active, and can be oxidized under proper conditions.

Tryptophan (2-amino-3-(1H-indol-3-yl)-propionic acid, TRP), is one of the twenty essential amino acids in the human diet (12). Tryptophan cannot be synthesized directly in the human body but must be absorbed tryptophan from food products and pharmaceutical formulas (13). This compound is mostly available in chocolates, egg, and milk. The common side effects in high dosage of tryptophan are drowsiness, nausea, dizziness, and loss of appetite. Tryptophan involves two main catabolic routes. In tryptamine pathway, it gives 5-hydroxy-tryptophan in the presence of tryptophan hydroxylase (14). The 5-hydroxytryptophan further gives the important neurotransmitter serotonin, which can affect the sleep, mood and mental health (15). Subsequently, serotonin has been converted into a neurohormone melatonin, which is used to improve sleep. Thus, the brain serotonin level depends on the tryptophan level in our body (16). Therefore, a rapid, simple, sensitive, and low cost detection method for tryptophan is of great interest. Different methods such as spectrophotometry, high performance liquid chromatography (HPLC), and electrochemical methods have so far been available for the determination of tryptophan (17-19). Among these methods, electrochemical methods have attracted more attention in two decades for food and biological compounds analysis due to quick response, low detection limit, low cost, simple operation, and the absence of pretreatment (20).

Overdose consumption of acetaminophen can alter tryptophan metabolism by inhibiting tryptophan 2, 3-dioxygenase activity thus increasing the availability of tryptophan for the production of serotonin in brain (21). Thus, the simultaneous determination of acetaminophen and tryptophan compounds could be of considerable value.

The screen-printed electrodes have been designed especially for miniaturization of electrochemical analytical systems (22, 23). SPEs are highly-versatile, easy to use, cost-effective analytical tools, also suitable to miniaturization (24). Furthermore, a screen printed electrode avoids the cleaning process, unlike conventional electrodes such as a glassy carbon electrode (GCE) (25, 26). In order to improve their electrochemical performance, SPEs have been modified with nano sized materials (27). The modified electrode has good electro catalytic activity, sensitivity, and selectivity; it has also a low detection limit compared to unmodified electrodes (28-32).

Fe_3_O_4_ nanoparticles have been extensively used for the modification of electrodes because they increase the electrode surface and electrical conductivity as well as improving electron transfer kinetics between the electrode surface and wide range of electroactive species (33, 34). Usually, an inert silica (SiO_2_) nanoparticle coating on the surface of magnetite nanoparticles prevents their aggregation, improves their chemical stability, and provides better protection against toxicity. The silica coating stabilizes the magnetite nanoparticles in two different ways, one of which is by shielding the magnetic dipole interaction with the silica shell. On the other hand, the silica nanoparticles are negatively charged. Therefore, the silica coating enhances the coulomb repulsion of the magnetic nanoparticles (35). 

Graphene, a single layer of sp^2^-hybrirdized carbon atoms packed in a honeycomb crystal lattice, has attracted considerable attention in recent years due to unique physical and chemical properties to be obtained. Graphene oxide (GO) is one of the most important derivatives of graphene having large surface area, excellent conductivity, and strong mechanical strength and contains abundant C–O–C (epoxide), C–OH, and terminated COOH groups decoration of GO by magnetic iron oxide (e.g., maghemite γ-Fe_2_O_3_ or magnetite Fe_3_O_4_) nanoparticles, which are very important magnetic materials, can improve optical, magnetic and electrochemical properties of GO. These properties make GO a great candidate for electrode surface modification (37, 38).

There are few reports of the use of an electrochemical sensor for the simultaneous determination of acetaminophen and tryptophan compounds. In this work, we introduce the simple application of a GO/Fe_3_O_4_@SiO_2 _nanocomposite modified graphite screen printed electrode as a sensitive sensor for this purpose. The proposed sensor showed good electrocatalytic effect on acetaminophen. Eventually, we evaluate the analytical performance of the suggestion sensor for acetaminophen and tryptophan determination in drug and urine samples.

## Experimental

*Apparatus and chemicals*

The electrochemical measurements were performed with an Autolab potentiostat/galvanostat (PGSTAT 302N, Eco Chemie, the Netherlands). The experimental conditions were controlled with General Purpose Electrochemical System (GPES) software. The screen-printed electrode (DropSens, DRP-110, Spain) consists of three main parts which are a graphite counter electrode, a silver pseudo-reference electrode and, a graphite working electrode.

All solutions were freshly prepared with double distilled water. Acetaminophen, tryptophan, and all other reagents were of analytical grade and were obtained from Merck chemical company (Darmstadt, Germany). The buffer solutions were prepared from orthophosphoric acid and its salts in the pH range of 2.0-9.0. 

For real samples analysis acetaminophen tablets and acetaminophen oral solution were purchased from Aria Pharmaceutical Company, Iran, and the urine samples were collected from a healthy person. 

*Synthesis of GO/Fe*_3_ *O*_4_ *@SiO*_2_ *nanocomposite*

For carboxylation of GO, an aqueous suspension (50 mL) of GO was diluted by a factor of 2 to give a concentration of 2 mg mL^−1^, and then bath sonicated for 1 h to give a clear solution. NaOH (12 g) and chloroacetic acid (Cl–CH_2_–COOH) (10 g) were added to the GO suspension and bath sonicated for 2 h to convert the –OH groups to –COOH via conjugation of acetic acid moieties giving G-COOH. The resulting G-COOH solution was neutralized, and purified by repeated rinsing and filtration.

About 0.06 g of GO–COOH was dissolved in 42 mL of water by ultrasonic irradiation (Sono swiss SW3-H, 38 kHz, Switzerland) for 20 min. The mixture was further stirred vigorously for 30 min at 60 °C. Then 106.2 mg of FeCl_3_·6H_2_O was added under stirring. After the mixture was stirred vigorously for 30 min under N_2_ atmosphere, 57 mg of FeSO_4_·7H_2_O was added and keeping it stirred under N_2_ atmosphere for 30 min. At last 18 mL of 6% NH_4_OH aqueous solution was added into the mixture drop by drop at 60 °C during 1 h and reacted for another 2 h. N_2_ atmosphere was used during the reaction to prevent critical oxidation. The reaction mixture was then centrifuged, washed with double distilled water, and dried. The obtained black precipitate was GO/Fe_3_O_4_ nanoparticle and was ready for use. Core–shell GO/Fe_3_O_4_@SiO_2 _nanocomposites were prepared by growing silica layers onto the surface of the GO/Fe_3_O_4_ as described by Lu *et al*. Fifteen milliliters of ethanol, 0.6 mL water, 0.6 mL ammonium hydroxide and 90 μL of TEOS were added in a 250 mL three neck flask in a 40 °C water bath. GO/Fe_3_O_4_ was added to the above solution under mechanical stirring. Aliquots of the mixture were taken out after 12 h by centrifugation and washed with water and vacuum-dried at 60 °C overnight. 

*Preparation of the electrode*

The bare screen-printed electrode was coated with GO/Fe_3_O_4_@SiO_2_ as follows. A stock solution of GO/Fe_3_O_4_@SiO_2 _in 1 mL aqueous solution was prepared by dispersing 1 mg GO/Fe_3_O_4_@SiO_2 _with ultrasonication for 1 h, and a 5 µL aliquot of the GO/Fe_3_O_4_@SiO_2 _/H_2_O suspension solution was casted on the carbon working electrodes, and waiting until the solvent was evaporated in room temperature. 

*Preparation of real samples*

Five acetaminophen tablets (labeled 325 mg per tablet, Aria Pharmaceutical Company, Iran) were grinding. Then, the tablet solution was prepared by dissolving 325 mg of the powder in 25 mL water by ultrasonication. Then, different volume of the diluted solution was transferred into a 25 mL volumetric flask and diluted to the mark with PBS (pH 7.0). The acetaminophen content was analyzed by the proposed method using the standard addition method.

The acetaminophen oral solution was diluted 100 times with water; then, different volume of the diluted solution was transferred into a 10 mL volumetric flask and diluted to the mark with PBS (pH 7.0). The acetaminophen content was analyzed by the proposed method using the standard addition method.

Urine samples were stored in a refrigerator immediately after collection. 10 mL of the sample was centrifuged for 15 min at 2000 rpm. The supernatant was filtered out using a 0.45 μm filter. Then, different volume of the solution was transferred into a 25 mL volumetric flask and diluted to the mark with PBS (pH 7.0). The diluted urine sample was spiked with different amounts of acetaminophen and tryptophan.

## Results and Discussion

*Electro-oxidation of acetaminophen at a GO/Fe*_3_ *O*_4_ *@SiO*_2_ */SPE*

**Scheme 1:**
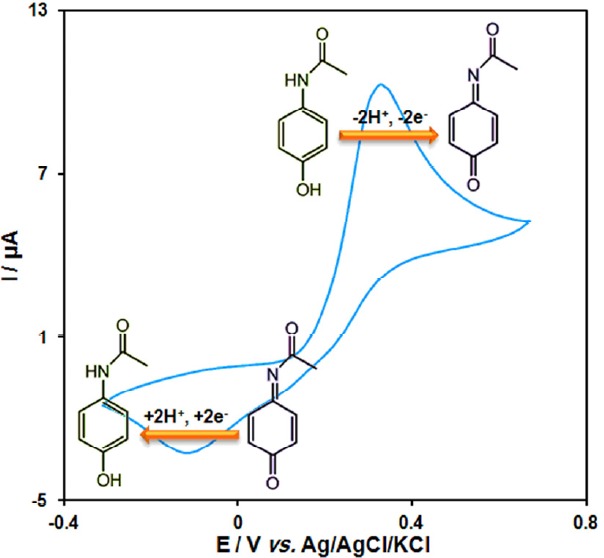
Transformation of acetaminophen to N-acetyl-p-benzoquinone-imine and vice-versa within a quasi-reversible two-electron process

Scheme 1 shows a cyclic voltammogram was recorded for acetaminophen in 0.1 M PBS (pH = 7.0). The voltammogram shows an anodic peak in the positive-going scan and a cathodic counterpart peak in the negative-going scan which corresponds to the transformation of acetaminophen to N-acetyl-p-benzoquinone-imine and vice-versa within a quasi-reversible two-electron process.

The electrochemical behavior of acetaminophen is dependent on the pH value of the aqueous solution. Therefore, pH optimization of the solution seems to be necessary in order to obtain the best results for electro-oxidation of acetaminophen. Thus, the electrochemical behavior of acetaminophen was studied in 0.1 M PBS in different pH values (2.0-9.0) at the surface of GO/Fe_3_O_4_@SiO_2_/SPE by voltammetry. It was found that the electro-oxidation of acetaminophen at the surface of GO/Fe_3_O_4_@SiO_2_/SPE was more favored under neutral conditions than in acidic or basic medium. This appears as a gradual growth in the anodic peak for oxidation current of acetaminophen at the surface of GO/Fe_3_O_4_@SiO_2_/SPE. Thus, the pH 7.0 was chosen as the optimum pH for electro-oxidation of acetaminophen at the surface of GO/Fe_3_O_4_@SiO_2_/SPE.

**Figure 1:**
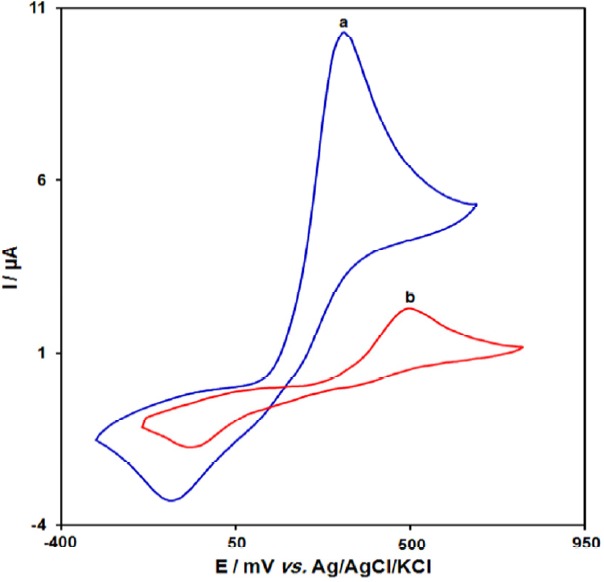
Cyclic voltammograms of (a) GO/Fe3O4@SiO2/SPE and (b) bare SPE in 0.1 M PBS (pH 7.0) in the presence of 100.0 μM acetaminophen at the scan rate 50 mVs-1

Figure 1 depict the cyclic voltammetric responses for the electrochemical oxidation of 100.0 μM acetaminophen at GO/Fe_3_O_4_@SiO_2_/SPE (curve a) and bare SPE (curve b). The anodic peak potential for the oxidation of acetaminophen at GO/Fe_3_O_4_@SiO_2_/SPE (curve a) is about 330 mV compared with 500 mV for that on the bare SPE (curve b). Similarly, when the oxidation of acetaminophen at the GO/Fe_3_O_4_@SiO_2_/SPE (curve a) and bare SPE (curve b) are compared, an extensive enhancement of the anodic peak current at GO/Fe_3_O_4_@SiO_2_/SPE relative to the value obtained at the bare SPE (curve b) is observed. In other words, the results clearly indicate that the combination of graphene and Fe_3_O_4_@SiO_2_ nanocomposites improve the acetaminophen oxidation signal.

**Figure 2:**
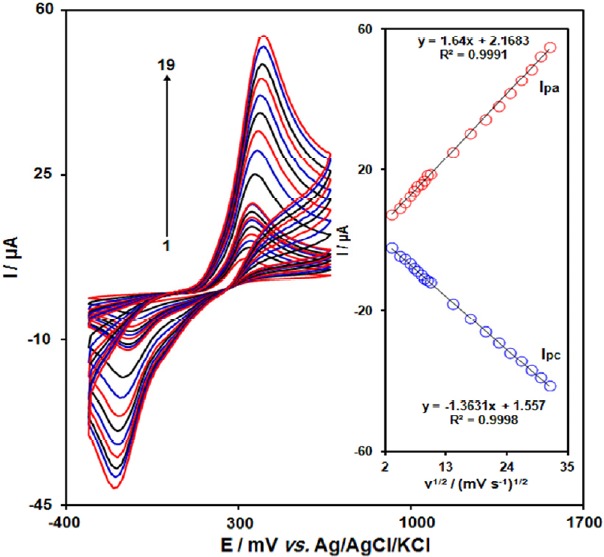
Cyclic voltammograms of GO/Fe3O4@SiO2/SPE in 0.1 M PBS (pH 7.0) containing 100.0 μM acetaminophen at various scan rates; numbers 1-19 correspond to 10, 20, 30, 40, 50, 60, 70, 80, 90, 100, 200, 300, 400, 500, 600, 700, 800, 900 and 1000 mV s-1, respectively. Inset: Variation of anodic and cathodic peak current vs. ν1/2

The effect of potential scan rates on the oxidation current of acetaminophen has been studied (Figure 2). The results showed that increasing in the potential scan rate induced an increase in the peak current. In addition, the oxidation process is diffusion controlled as deduced from the linear dependence of the anodic peak current (I_p_) on the square root of the potential scan rate (ν^1/2^) over a wide range from 10 to 1000 mV s^−1^.

**Figure 3:**
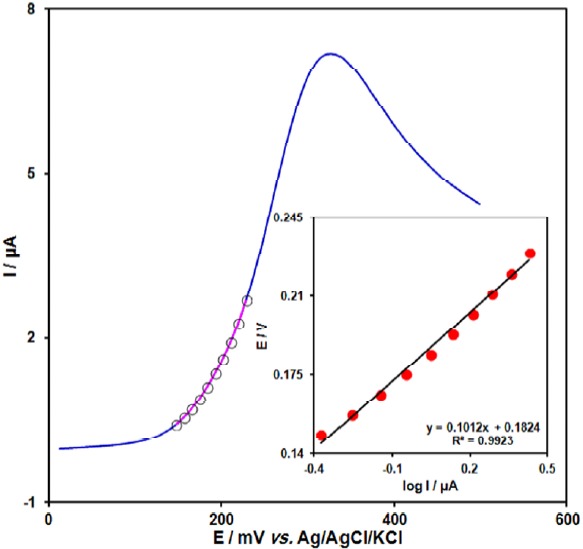
LSV (at 10 mV s−1) of electrode in 0.1 M PBS (pH 7.0) containing 100.0 µM acetaminophen. The points are the data used in the Tafel plot. The inset shows the Tafel plot derived from the LSV

Figure 3 shows a Tafel plot that was drawn from points of the Tafel region of the linear sweep voltammetry (LSV). The Tafel slope of 0.1012 V obtained in this case agrees well with the involvement of one electron in the rate determining step of the electrode process, assuming a charge transfer coefficient of α = 0.42 (39).

**Table 1:** Comparison of the efficiency of some methods used in detection of acetaminophen

**Method**	**Modifier**	**LOD**	**LDR**	**Ref.**
Voltammetry	Au nanoparticles/poly(caffeic acid)	14 nM	0.2-1000.0 μM	(40)
Voltammetry	Co(II)-exchanged zeolite A (Co(II)-A/ZMCPE)	0.04 μM	0.1-190.0 μM	(41)
Voltammetry	Graphene nanoplatelets like structures formation on ionic liquid/carbon-ceramic	63 nM	0.1-20.0 μM	(42)
Voltammetry	PbS nanoparticles Schiff base	5.36 nM	0.033-158.0 μM	(43)
Voltammetry	Gold nanoparticles, functionalized multi-walled carbon nanotubes and cobalt (II) phthalocyanine	0.135 μM	1.49-107.0 μM	(44)
Voltammetry	Poly overoxidized pyrrole–aszophloxine–gold	0.08 μM	0.2-100.0 μM	(45)
Voltammetry	Graphene oxide/Fe3O4@SiO2 nanocomposite	0.1 μM	0.5-100.0 μM	This work

**Table 2:** The application of GO/Fe3O4@SiO2/SPE for simultaneous determination of acetaminophen and tryptophan in acetaminophen tablet, acetaminophen oral solution and urine samples (n = 5*). All concentrations are in µM

**Sample ** ** Spiked**				**Found**			**Recovery (%)**			**R.S.D. (%)**		
Acetaminophen tablet	Acetaminophen	Tryptophan	Acetaminophen		Tryptophan	Acetaminophen		Tryptophan	Acetaminophen		Tryptophan
	0	0	10.0		-	-		-	2.3		-
	2.5	10.0	12.9		9.9	103.2		99.0	3.3		2.8
	5.0	20.0	14.8		20.2	98.7		101.0	1.7		3.1
Acetaminophen oral solution	0	0	12.0		-	-		-	3.2		-
	5.0	15.0	17.5		14.7	102.9		98.0	2.9		1.7
	10.0	25.0	21.8		25.8	99.1		103.2	2.4		3.2
	0	0	-		-	-		-	-		-
**Urine**	10.0	12.5	9.9		12.8	99.0		102.4	1.9		2.9
	15.0	17.5	15.5		17.3	103.3		98.9	3.0		2.2

* Out of five experiments

**Figure 4:**
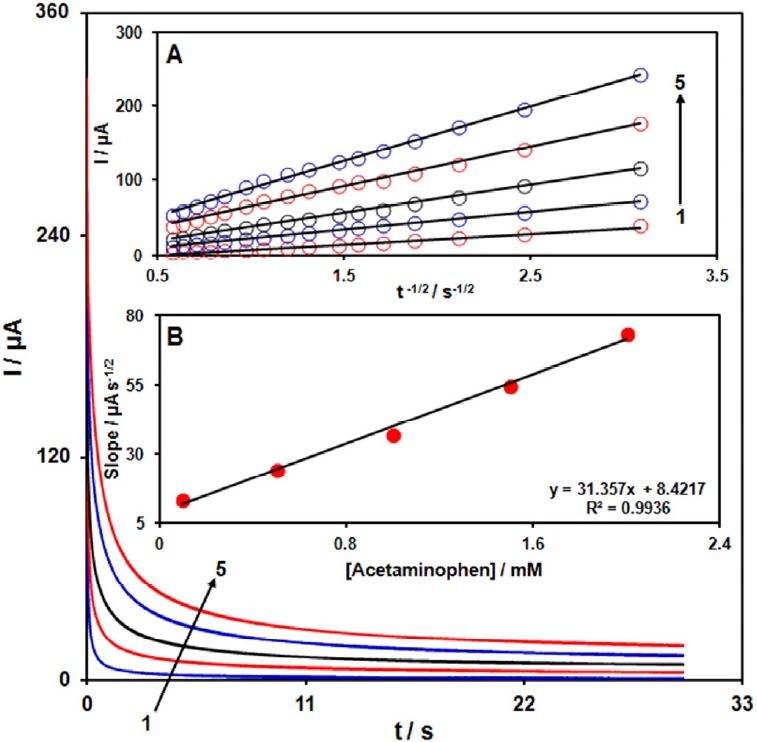
Chronoamperograms obtained at GO/Fe3O4@SiO2/SPE in 0.1 M PBS (pH 7.0) for different concentration of acetaminophen. The numbers 1–5 correspond to 0.1, 0.5, 1.0, 1.5 and 2.0 mM of acetaminophen. Insets: (A) Plots of I vs. t-1/2 obtained from chronoamperograms 1–5. (B) Plot of the slope of the straight lines against acetaminophen concentration

**Figure. 5:**
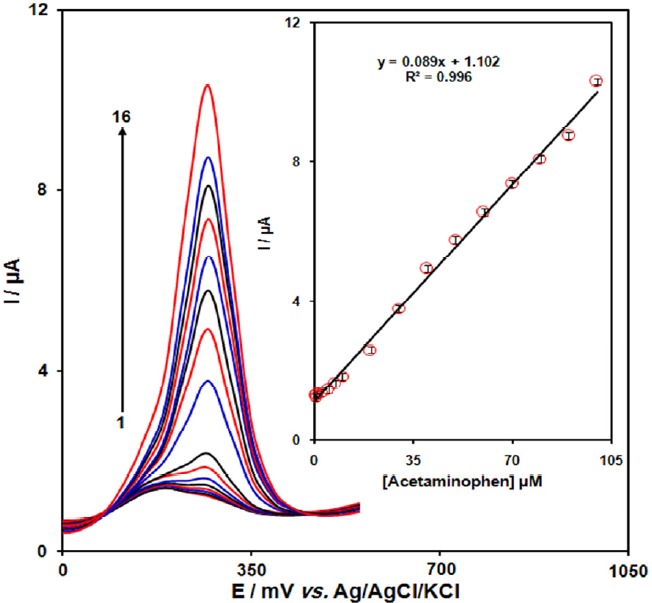
DPVs of GO/Fe3O4@SiO2/SPE in 0.1 M (pH 7.0) containing different concentrations of acetaminophen. Numbers 1–16 correspond to 0.5, 0.75, 1.0, 2.5, 5.0, 7.5, 10.0, 20.0, 30.0, 40.0, 50.0, 60.0, 70.0, 80.0, 90.0 and 100.0 µM of acetaminophen. Inset: plot of the electrocatalytic peak current as a function of acetaminophen concentration in the range of 0.5-100.0 µM

**Figure. 6:**
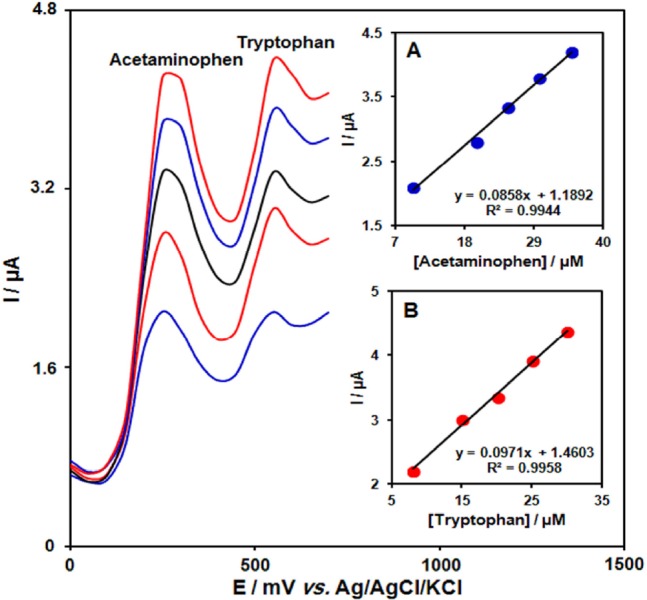
DPVs of GO/Fe3O4@SiO2/SPE in 0.1 M PBS (pH 7.0) containing different concentrations of acetaminophen + tryptophan in μM, from inner to outer: 10.0+8.0, 20.0+15.0, 25.0+20.0, 30.0+25.0 and 35.0+30.0 respectively. Insets: (A) plot of Ip *vs. *acetaminophen concentrations and (B) plot of Ip vs. tryptophan concentrations

*Chronoamperometric measurements*

Chronoamperometric measurements of acetaminophen at GO/Fe_3_O_4_@SiO_2_/SPE were carried out by setting the working electrode potential at 0.4 V for the various concentration of acetaminophen in PBS (pH 7.0) (Figure 4). For an electroactive material (acetaminophen in this case) with a diffusion coefficient of D, the current observed for the electrochemical reaction at the mass transport limited condition is described by the Cottrell equation (39).

I =nFAD^1/2^C_b_π^-1/2^t^-1/2                                   ^Equ. 1

Where D and C_b_ are the diffusion coefficient (cm^2^ s^-1^) and the bulk concentration (mol cm^−3^), respectively. Experimental plots of I vs. t^−1/2^ were employed, with the best fits for different concentrations of acetaminophen (Figure. 4A). The slopes of the resulting straight lines were then plotted vs. acetaminophen concentration (Figure 4B). From the resulting slope and Cottrell equation the mean value of the D was found to be 8.39 × 10^−5^ cm^2^/s. 

*Calibration plot and limit of detection*

The peak current of acetaminophen oxidation at the surface of the modified electrode can be used for determination of acetaminophen in solution. Therefore, differential pulse voltammetry (DPV) experiments were done for different concentrations of acetaminophen. The oxidation peak currents of acetaminophen at the surface of a modified electrode were proportional to the concentration of the acetaminophen within the ranges 0.5 to 100.0 μM. The detection limit (3σ) of acetaminophen was found to be 0.1 µM. These values are comparable with values obtained by other research groups (Table 1).

*Simultaneous determination of acetaminophen and tryptophan*

Determination of two compounds was performed by simultaneously changing the concentrations of acetaminophen and tryptophan, and recording the DPVs (Figure 6). The voltammetric results showed well-defined anodic peaks at potentials of 250 and 550 mV, corresponding to the oxidation of acetaminophen and tryptophan, respectively, indicating that simultaneous determination of these compounds is feasible at the GO/Fe_3_O_4_@SiO_2_/SPE.

The sensitivity of the modified electrode towards the oxidation of acetaminophen was found to be 0.0858 µA µM^-1^, which is very close to the value obtained in the absence of acetaminophen and tryptophan (0.0892 µA µM^-1^) (Figure 6). These results are indicative that the oxidation processes of these compounds at the GO/Fe_3_O_4_@SiO_2_/SPE are independent and therefore, simultaneous determination of their mixtures is possible without significant interferences. 

*Real sample analysis*

In order to evaluate the analytical applicability of the proposed method, also it was applied to the determination of acetaminophen and tryptophan in acetaminophen tablet, acetaminophen oral solution and urine samples. The results for determination of these species in real samples are given in Table 2. Satisfactory recovery of the experimental results was found for acetaminophen and tryptophan. The reproducibility of the method was demonstrated by the mean relative standard deviation (R.S.D.).

## Conclusions

A GO/Fe_3_O_4_@SiO_2_ nanocomposite modified screen printed electrode was successfully fabricated. Compared with bare SPE, the voltammetric responses of GO/Fe_3_O_4_@SiO_2_/SPE were significantly enhanced. The reason is the increase of the electrochemical active surface area and the conductivity of the electrode by introducing GO/Fe_3_O_4_@SiO_2_ nanocomposite. Under optimum condition, the peak current was proportional to the acetaminophen concentration in the range of 0.5–100.0 µM with the detection limit of 0.1 µM. Additionally, the GO/Fe_3_O_4_@SiO_2_/SPE exhibited an excellent electrocatalytic activity for acetaminophen and tryptophan in biological and pharmaceutical samples.
